# ACR benchmark testing of a novel high‐speed ring‐gantry linac kV‐CBCT system

**DOI:** 10.1002/acm2.14299

**Published:** 2024-03-22

**Authors:** Allison Haertter, Michael Salerno, Brandon Koger, Christopher Kennedy, Michelle Alonso‐Basanta, Lei Dong, Boon‐Keng Teo, Taoran Li

**Affiliations:** ^1^ Department of Radiation Oncology University of Pennsylvania Philadelphia Pennsylvania USA

**Keywords:** ACR, CBCT, treatment planning

## Abstract

A new generation cone‐beam computed tomography (CBCT) system with new hardware design and advanced image reconstruction algorithms is available for radiation treatment simulation or adaptive radiotherapy (HyperSight CBCT imaging solution, Varian Medical Systems‐a Siemens Healthineers company). This study assesses the CBCT image quality metrics using the criteria routinely used for diagnostic CT scanner accreditation as a first step towards the future use of HyperSight CBCT images for treatment planning and target/organ delineations. Image performance was evaluated using American College of Radiology (ACR) Program accreditation phantom tests for diagnostic computed tomography systems (CTs) and compared HyperSight images with a standard treatment planning diagnostic CT scanner (Siemens SOMATOM Edge) and with existing CBCT systems (Varian TrueBeam version 2.7 and Varian Halcyon version 2.0).  Image quality performance for all Varian HyperSight CBCT vendor‐provided imaging protocols were assessed using ACR head and body ring CT phantoms, then compared to existing imaging modalities. Image quality analysis metrics included contrast‐to‐noise (CNR), spatial resolution, Hounsfield number (HU) accuracy, image scaling, and uniformity. All image quality assessments were made following the recommendations and passing criteria provided by the ACR. The Varian HyperSight CBCT imaging system demonstrated excellent image quality, with the majority of vendor‐provided imaging protocols capable of passing all ACR CT accreditation standards. Nearly all (8/11) vendor‐provided protocols passed ACR criteria using the ACR head phantom, with the Abdomen Large, Pelvis Large, and H&N vendor‐provided protocols produced HU uniformity values slightly exceeding passing criteria but remained within the allowable minor deviation levels (5–7 HU maximum differences). Compared to other existing CT and CBCT imaging modalities, both HyperSight Head and Pelvis imaging protocols matched the performance of the SOMATOM CT scanner, and both the HyperSight and SOMATOM CT substantially surpassed the performance of the Halcyon 2.0 and TrueBeam version 2.7 systems. Varian HyperSight CBCT imaging system could pass almost all tests for all vendor‐provided protocols using ACR accreditation criteria, with image quality similar to those produced by diagnostic CT scanners and significantly better than existing linac‐based CBCT imaging systems.

## 1 BACKGROUND

Standard diagnostic computed tomography (CT) images are acquired using fan‐beam geometry and slice‐by‐slice acquisition that is then reconstructed to produce full 3D volumetric images.^1^ Meanwhile, kV cone‐beam computed tomography (CBCT) images are acquired using a large angle cone‐beam geometry, capturing the entire 3D volumetric images, and performing 2D projection reconstructions.^2^, ^3^ The larger field of view (FOV) of CBCT images has several disadvantages, such as more significant amounts of scatter and noise,^2^, ^4^, ^5^ beam hardening,^2^, ^4^ detector nonlinearities,^2^, ^6^ and source intensity fluctuations.^7^ Additionally, CBCT images have been documented to be prone to crescent artifacts when using bowtie filters^2^, ^8^ and ring artifacts due to defective or malfunctioning detector elements.^2^ All of these disadvantages impact not only image quality but also the quantitative Hounsfield unit (HU) values produced by the image, which brings the reliability of CBCT‐based dose calculations into question.^4^ Currently, linear accelerator (linac) based CBCT imaging is utilized predominantly for daily patient positioning and for increased soft tissue and organ variation analysis compared to traditional kV or MV portal imaging^2^; however, the use of adaptive radiation therapy has recently expanded. The current integration of CBCT into adaptive radiotherapy has been primarily through either manual HU overrides within regions of large HU differences between the planning CT and daily CBCT imaging,^9^ synthetic CT generation from CBCT images,^10^, ^11^, ^12^ or through deformable registration of the planning CT onto the CBCT.^4^, ^13^ At this time, there is no CBCT‐based imaging system that has image quality rivaling that produced by traditional diagnostic CT simulators, and CBCT systems are not able to be used for accurate dose calculations alone without a corresponding CT simulator image. Fan‐beam CT as used in diagnostic CT simulators are accepted as the standard of care for radiotherapy treatment planning but are also known have to HU accuracy and uniformity differences compared to ground truth due to varying parameters or position within the scan field of view.

The newly introduced HyperSightTM system (Varian Medical Systems, Palo Alto, CA) is an upgraded CBCT imaging technology to allow for larger, faster, and enhanced in‐room imaging on the HalcyonTM and EthosTM version 4.0 systems (Varian Medical Systems, Palo Alto, CA). The Halcyon system allows for fast and accurate patient alignment and treatments,^14^, ^15^ with the HyperSight system available on Halcyon 4.0. HyperSight systems have an increased gantry rotation speed of 6 RPM, compared to 4 RPM on previous Halcyon and Ethos systems, and when combined with the HyperSight system's higher heat capacity kV x‐ray tube, high‐quality CBCT images can be acquired within 6 s. The HyperSight system has two modes of use: Cone Beam CT for Planning (CBCTp) and daily alignment imaging (IGRT mode). The CBCTp mode has a default slice thickness setting of 3 mm but allows for different thickness selections (1, 2, 3, and 5 mm), allows for higher default imaging doses, and restricts kVp use to only 125 and 140 kVp. The acquisition speeds in the default protocols vary between 6 and 60 s (6, 10, and 60 s) to allow elevated imaging dose or thoracic volume‐averaged imaging. The IGRT mode allows only 1 slice thickness (2 mm), has reduced default imaging doses, and permits 80 and 100 kVp use for further imaging dose reductions.

In addition to an increase in gantry rotation speed, the HyperSight system includes imaging hardware and algorithm upgrades to allow for high quality kV CBCT imaging. Imaging hardware upgrades include a high‐capacity kV x‐ray tube, a larger imaging panel (86 cm × 43 cm), a movable bowtie filter, and kV collimator system. Algorithm upgrades include an iterative reconstruction algorithm (iCBCT) with additional motion and metal artifact reduction, scatter reduction with Acuros CTS and Monte Carlo‐modeled hardware scatter, extended FOV reconstruction, and energy‐based HU calibrations which produce CBCT images of excellent quality and HU accuracy for dose calculation. The kV imager includes a lead anti‐scatter grid^14^ and a new 2‐step iCBCT algorithm, which is superior to the standard Feldkamp Davis Kress (FDK) CBCT reconstruction algorithm,^2^ resulting in reduced levels of noise and scatter. The Acuros CTS reconstruction algorithm implemented is a linear Boltzmann‐based algorithm that estimates projection‐specific scatter components that are then subtracted from each projection image to reduce the overall level of scatter within the CBCT image.^19^ Monte‐Carlo modeled hardware scatter reductions also reduce image noise and scatter, and when combined with the additional energy spectrum‐dependent HU calibration, provides increased HU uniformity and accurate material HU values regardless of imaging protocol energy (125 or 140 kVp).

**FIGURE 1:**
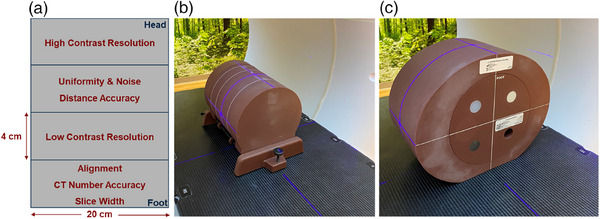
ACR CT head and body ring design and setup. (a) ACR head phantom module design and size, (b) head phantom setup, (c) body ring and head phantom setup.

The American College of Radiology (ACR) is a peer‐reviewed and self‐assessed accreditation process for various imaging modalities, with the goal of assessing an institution's imaging equipment, imaging dose, and image quality. ACR accreditation is viewed as a quality standard metric to ensure good imaging quality and practices for both radiology and radiation oncology departments.^16^ For CT accreditation, a specialized phantom is provided for head imaging protocol testing and an attachable body ring is provided for body imaging protocol testing, both of which can be visualized in Figure 1. The ACR provides structured guidelines on passing criteria for various treatment sites, such as head, body, pediatric head, and pediatric body, for the image quality parameters of positioning accuracy, HU number accuracy, slice width, contrast‐to‐noise ratio (CNR), spatial resolution, image uniformity, and scaling.^17^ The HyperSight system is the first linac‐based kV CBCT system to offer treatment planning quality CBCT images. To assess the image quality and to provide a baseline of the HyperSight system's image performance, the system was subjected to the ACR diagnostic CT simulator quality control assessment. This study is the first to report on the imaging performance of the HyperSight system, evaluating the system with the same ACR CT quality standard metrics that many diagnostic CT units are subjected to and comparing its image quality to pre‐existing kV CBCT and diagnostic CT imaging systems.

## 2 METHODS

The ACR CT phantom (Figure 1) is composed of a head phantom (20 cm × 16 cm) and an optional outer body ring (20 cm × 33 cm) for abdomen, thorax, and pelvis image protocol testing. The head phantom contains four modules to test various image quality parameters, such as CNR, uniformity, noise, distance accuracy, spatial resolution, HU number accuracy, and scan slice width (Figure 1).^18^ CNR is assessed by comparing the ROI HU average and standard deviation between two different HU ROIs using Equation (1):

(1) $$CNR=\frac{\left|ROI\;A\;average-ROI\;B\;average\right|}{Standard\;Deviation\;of\;ROI\;B}$$

HU uniformity is evaluated by placing four peripheral and one center ROI and comparing the mean ROI HU difference of each peripheral ROI to that of the center ROI. Placing two metal ball bearings at a known distance apart allows for image scaling and distance accuracy assessments. Spatial resolution can be assessed by the visible line pairs/cm from eight bar patterns placed within the phantom. HU number accuracy includes testing of polyethylene, bone, acrylic, air, and water, with specified HU bounds of passing, minor, and major deviations recommended by the ACR.

Within this study, the ACR head and body ring phantoms were scanned using all vendor‐provided imaging protocols (11 in total) using the CBCTp mode, using the setup shown in Figure 1A and B. The head phantom was placed on the ACR provided phantom stand with 4 cm solid water slabs placed on both ends of the phantom to provide additional scatter (Figure 1A). The body ring and head phantom were scanned, as shown in Figure 1B, with supplemental scattering materials placed on both sides of the phantom to provide additional scattering materials. Both phantoms were placed at a medial location on the treatment couch, with the phantom midlines aligned with the machine laser isocentering systems. Prior to CBCTp imaging on the HyperSight system, topogram images were acquired to ensure correct alignment and adequate kV blade openings. For all imaging protocols, the nominally Varian provided default imaging quality parameters, such as kVp, mAs, and volume computed tomography dose index (CTDI_vol_), were used, which are shown in Table 1. All CBCTp imaging protocols were acquired using 3 mm and also reconstructed to 2 mm slices.

**TABLE 1:** HyperSight CBCTp and machine comparison (Siemens SOMATOM Edge, Varian TrueBeam, and Varian Halcyon 2.0) vendor‐provided imaging protocol parameters.

Machine	Imaging protocol	Energy (kV)	Exposure (mAs)	CTDI_vol_ (mGy)
**HyperSight (Ring gantry LINAC)**				
	Head	125	806.2	34.5
	H&N	125	703.6	30.1
	Thorax	125	703.6	13.4
	Thorax Slow^a^	125	703.1	13.4
	Breast	125	409.0	7.8
	Abdomen	125	527.7	9.95
	Abdomen large	140	873.7	22.3
	Pelvis	125	527.7	9.95
	Pelvis large	140	1005.0	25.7
	Pediatric head	125	409.0	17.6
	Pediatric abdomen	125	146.5	2.76
**Siemens SOMATOM edge (Diagnostic CT)**				
	Head	120	240	16.1
	Abdomen	120	280	18.8
	Pelvis	120	280	18.8
	Pediatric head	120	120	8.08
	Pediatric abdomen	120	100	6.74
**Varian TrueBeam (C‐Arm LINAC)**				
	Head	125	150	3.17
	Pelvis	100	4050	60.1
**Varian Halcyon 2.0 (Ring Gantry LINAC)**				
	Head	100	100	3.33
	Pelvis	125	1080	21.6
	Pelvis large	140	1440	38.02

^a^ HyperSight Thorax Slow scan is a 60‐s image acquisition (compared with a 6‐s acquisition for Thorax) in an attempt to blur out breathing motion artifacts for the ITV‐based treatment technique.

**FIGURE 2:**
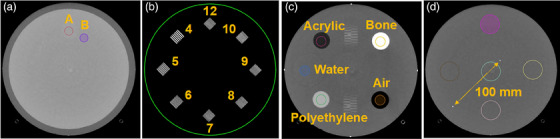
ACR head phantom module analysis. (a) CNR module analysis ROIs, (b) spatial resolution bar patterns, (c) HU accuracy ROI placements, (d) uniformity ROIs and image scaling measurement location.

**FIGURE 3:**
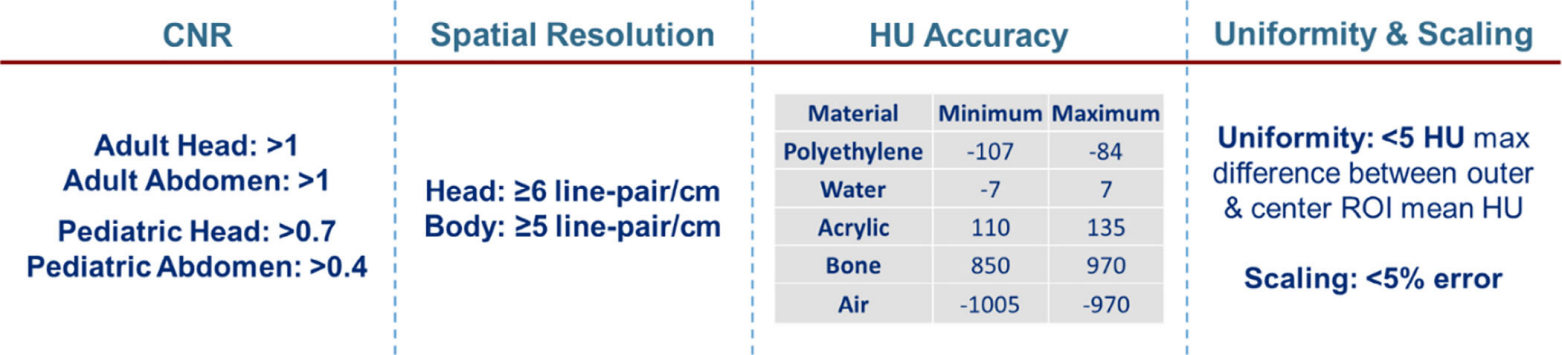
ACR phantom passing criteria for CNR, spatial resolution, HU accuracy, uniformity, and image scaling based on imaging protocol selected.

Image analysis was performed according to ACR specifications, which can be found online,^18^ with the ROI delineation for all image analyses shown in Figure 2. CNR was evaluated by placing ROIs in two varying HU regions and inputting the average HU within each ROI region and the HU standard deviation within ROI B into Equation 1 (Figure 2A). Eight bar pattern regions are provided within the ACR phantom, ranging from 4 to 12 line‐pairs/cm to quantify spatial resolution (Figure 2B). HU accuracy was evaluated by placing ROIs in the center of varying HU materials and recording the mean HU within the ROI (Figure 2C) compared to the ACR defined maximum and minimum passing HU values for each material. Five equally sized ROIs were placed within a uniform module slab of the phantom, with four placed along the periphery and one within the center (Figure 2D), with the difference in average HU between each peripheral ROI and the center ROI calculated to assess HU uniformity. Two metal ball bearings are spaced 100 mm apart and measured on each image (Figure 2D) to quantify image scaling. The ACR provides passing criteria for each module, with non‐imaging protocol specific passing criteria for HU accuracy, uniformity, scaling, and imaging protocol‐specific passing criteria for CNR and spatial resolution, which are provided in Figure 3. Current ACR recommendations do not provide high contrast spatial resolution passing criteria for CT machines, but previous ACR recommendations stated passing criteria of at least six line‐pairs/cm for head protocols and at least five line‐pairs/cm for body protocols. Within this study, these previous ACR spatial resolution recommendations were used for investigational image quality purposes. For uniformity analysis, the ACR quantifies < 5 HU maximum difference to be passing, and 5−7 HU maximum difference to be a minor deviation.

As the HyperSight system is marketed to be of similar image quality to that of a traditional diagnostic CT simulator while still being a linac‐based CBCT imaging system, the same ACR phantoms and quality criteria were used on a pre‐existing diagnostic CT simulator and two common linac‐based CBCT imaging systems and used as a benchmark comparison to the HyperSight system. For the diagnostic CT simulator comparison, a Siemens SOMATOM Edge (Siemens Healthineers, Erlangen, Germany) was used, and for the linac‐based CBCT imaging systems, a Varian TrueBeam 2.7 and Varian Halcyon version 2.0 were used. Six HyperSight default imaging protocols were tested (Head, Abdomen, Pelvis, Pelvis Large, Pediatric Head, and Pediatric Abdomen) using 2 mm slice thickness, with the ACR phantom chosen according to the ACR recommended phantom for each imaging protocol (head and pediatric protocols using the head phantom and pelvis/abdomen protocols using the body phantom). For the comparison machines, SOMATOM Edge, TrueBeam 2.7, and Halcyon 2.0, any matching default imaging protocols were tested using 2 mm slices and the same ACR phantom used for the HyperSight testing. The imaging protocol parameters used for the machine comparison can be found in Table 1.

The ACR head and body phantoms were then scanned several months later as a check on the reproducibility of the HU accuracy of the HyperSight system. The Head, Pelvis, Pelvis Large, and Breast imaging protocols were scanned with the same default imaging parameters previously used (Table 1). The ACR phantom selected for each protocol was based on the phantom size recommended by the ACR guidelines, with the head phantom used for the Head protocol and the body phantom used for the Pelvis, Pelvis Large, and Breast protocols. All analyzed images had a slice thickness of 3 mm, with the same ACR phantom being used for analysis between the original and repeated scans. The reproducibility analysis contained only the CT number accuracy of acrylic, air, bone, polyethylene, and water.

## 3 RESULTS

### 3.1 HyperSight image quality performance

ACR passing scores for all vendor‐provided default HyperSight CBCTp mode imaging protocols for both the head and body phantoms are shown in Table 2. Green colored boxes represent passing ACR criteria, yellow representing minor deviations from ACR criteria, and red representing major deviation from ACR criteria. The head phantom results show the HyperSight system produces images of excellent image quality, with Figure 2 showing a slice of each ACR phantom module produced by the HyperSight Head imaging protocol. All protocols scanned with the ACR head phantom were capable of passing ACR criteria for contrast resolution, spatial resolution, CT number accuracy, and image scaling (Table 2). Three imaging protocols (H&N, Abdomen Large, and Pelvis Large) produced minor deviations in HU uniformity by having maximum HU differences of < 7 HU.  When assessing ACR evaluations on the ACR body phantom, no imaging protocol passed all criteria due to significant attenuation within the large phantom; however, all protocols passed the spatial resolution, polyethylene HU accuracy, and image scaling ACR criteria (Table 2). The two imaging protocols designed for larger patient thicknesses with higher kVp (Abdomen Large and Pelvis Large) passed contrast resolution standards. With mAs optimization beyond the default values, other imaging protocols are likely to pass CNR criteria. 2/11 (18.2%) imaging protocols passed HU uniformity metrics, and 5/11 (45.5%) produced a minor deviation; 4/11 (36.4%) imaging protocols failed HU uniformity metrics with maximum HU differences from peripheral to center of < 10 HU. Further investigation on the CT number accuracy failing for acrylic, air, bone, and water materials showed that the average maximum difference between the HyperSight imaged and the ACR HU passing criteria is relatively low, with the average failing HU value for acrylic being 9.4 ± 4.8 HU, −40.6 ± 3.3 HU for air, 32.7 ± 28.6 HU for bone, and 7.2 ± 3.6 HU for water.

**TABLE 2:** ACR head phantom scores for all default HyperSight imaging protocols.

			CT number accuracy						
Imaging protocol	Contrast resolution	Spatial resolution (lp/cm)	Acrylic	Air	Bone	Polyethylene	Water	Image scaling	HU uniformity
**Head phantom**									
Head	2.5	6	115.8	‐999.2	931.3	‐100.0	‐3.1	0.6%	4.05
H&N	2.0	6	117.5	‐998.1	931.9	‐98.2	‐0.92	0.2%	5.10
Thorax	2.2	5	117.2	‐998.1	931.8	‐98.9	‐1.2	0.9%	4.02
Thorax slow	2.6	5	116.8	‐997.5	930.5	‐97.6	‐0.6	0.6%	3.66
Breast	1.4	5	116.5	‐997.4	928.1	‐97.7	‐0.7	0.2%	4.98
Abdomen	2.0	5	116.9	‐998.6	931.7	‐98.7	‐1.4	0.2%	3.93
Abdomen large	2.6	5	115.2	‐999.0	887.2	‐98.6	‐4.7	0.6%	6.05
Pelvis	1.7	5	116.5	‐998.9	931.3	‐99.8	‐2.0	0.5%	4.02
Pelvis large	3.3	5	115.1	‐999.3	887.2	‐98.7	‐5.4	0.2%	6.05
Pediatric head	1.5	6	117.5	‐999.9	938.2	‐95.7	‐0.2	0.4%	4.40
Pediatric abdomen	0.7	5	118.8	‐999.1	936.2	‐95.4	‐0.2	0.7%	3.04
**Body phantom**									
Head	0.7	6	102.4	‐971.2	831.0	‐93.3	‐11.6	0.2%	5.66
H&N	0.5	6	103.8	‐971.4	831.4	‐96.8	‐11.3	0.2%	3.99
Thorax	0.4	5	102.0	‐970.3	829.5	‐95.6	‐12.1	0.5%	4.51
Thorax slow	0.7	5	108.8	‐978.2	846.5	‐93.5	‐6.9	0.5%	8.17
Breast	0.2	5	101.6	‐969.5	830.2	‐93.5	‐13.5	0.2%	5.67
Abdomen	0.4	5	102.3	‐973.7	833.1	‐95.0	‐12.0	0.5%	5.83
Abdomen large	1.2	5	92.6	‐961.2	762.2	‐99.5	‐20.3	0.1%	6.62
Pelvis	0.4	5	102.7	‐975.2	837.0	‐94.9	‐12.1	0.7%	5.66
Pelvis large	1.0	5	92.4	‐963.0	764.5	‐99.6	‐21.1	0.8%	7.65
Pediatric head	0.6	6	99.3	‐962.6	805.2	‐97.7	‐12.2	0.1%	8.7r5
Pediatric abdomen	0.3	5	98.8	‐965.6	820.0	‐95.3	‐15.3	0.2%	9.79

*Note*: All protocols using 3 mm slice thicknesses (green = meeting ACR criteria, yellow = minor deviation from ACR criteria, red = major deviation from ACR criteria).

To assess the impact of increasing mAs and imaging dose on image quality, the ACR head phantom was rescanned four times using the Pediatric Head and Pediatric Abdomen protocols, which historically use very low exposure settings to reduce pediatric imaging dose, with ACR metrics evaluated and compared. The results of this analysis are shown in Table 3. All exposure settings for both imaging protocols resulted in passing ACR criteria images, with contrast resolution increasing between the lowest and highest exposure setting as expected. Exposure settings have only modest influence on resultant CT number, and as expected, CT numbers between the various exposure settings stayed very stable; CT numbers varied < 1.3 HU between the same imaging protocol at different exposures and < 2 HU between different exposures and imaging protocols.

**TABLE 3:** ACR head phantom scores for Hypersight pediatric head and abdomen imaging protocols with varying exposure settings.

			CT number accuracy						
Estimated imaging dose (mGy)	Contrast resolution	Spatial resolution (lp/cm)	Acrylic	Air	Bone	Polyethylene	Water	Image scaling	HU uniformity
**Pediatric head imaging protocol**									
17.6	1.5	6	117.5	‐999.9	938.2	‐95.7	‐0.2	0.4%	4.40
21.2	1.9	6	117.9	‐999.9	939.3	‐96.2	0.2	0.2%	3.64
26.8	1.8	6	117.4	‐999.9	939.5	‐96.8	‐0.5	0.5%	3.85
31.7	2.6	6	117.4	‐999.9	938.4	‐96.9	‐0.5	0.2%	3.53
**Pediatric body imaging protocol**									
2.8	0.7	5	118.8	‐999.1	936.2	‐95.4	‐0.2	0.7%	3.04
3.7	1.3	5	118.5	‐999.5	936.5	‐95.9	0.7	0.2%	2.70
5.1	1.9	5	118.5	‐999.6	937.2	‐95.3	0.3	0.2%	4.17
7.0	1.7	5	117.7	‐999.7	937.4	‐96.0	0.4	0.2%	3.51

*Note*: All images acquired with 3 mm slice thicknesses (green = meeting ACR criteria, yellow = minor deviation from ACR criteria, red = major deviation from ACR criteria).

### 3.2 Machine comparison performance

**FIGURE 4:**
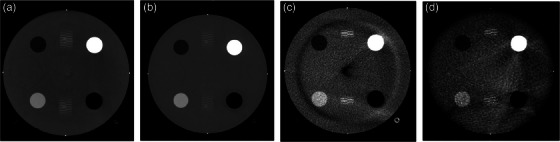
ACR head phantom HU accuracy module slice taken with window leveling set to pelvis (−160 to 240 HU) on the (a) Varian HyperSight, (b) Siemens SOMATOM Edge, (c) Varian TrueBeam 2.7, and (d) Varian Halcyon 2.0. All modalities using default Head imaging protocol.

Table 4 shows the machine comparison results of the ACR head and body phantom for 6 different default imaging protocols (Head, Abdomen, Pelvis, Pelvis Large, Pediatric Head, and Pediatric Abdomen) between the HyperSight imaging system, a diagnostic CT simulator (Siemens SOMATOM Edge), and two pre‐existing linac‐based CBCT imaging systems (Varian TrueBeam 2.7 and Varian Halcyon 2.0). Image quality using the default Head imaging protocol on all four machines can be visualized in Figure 4. For the Head and Pediatric Head imaging protocols utilizing the ACR head phantom, the HyperSight system matches the imaging quality and ACR passing criteria of the diagnostic SOMATOM Edge CT; however, the SOMATOM Edge scanner slightly outperforms the HyperSight system for the Abdomen, Pelvis and Pediatric Abdomen imaging protocols. HU uniformity was worse on all HyperSight images compared to the diagnostic CT, which can be expected due to its cone‐beam delivery and scatter effects. The HyperSight system comes with pre‐installed vendor‐provided imaging protocols, however protocols can be easily created, customized, and persisted for later use. Regarding the vendor‐provided imaging protocols imaging dose, the HyperSight system has a higher CTDI_vol_ than the diagnostic CT for only the Head and Pediatric Head imaging protocols, and lower CTDI_vol_ for Abdomen, Pelvis, and Pediatric Abdomen protocols (Table 1).

**TABLE 4:** ACR head phantom score comparison between diagnostic and existing linac‐based CBCT imaging systems.

				CT number accuracy						
Machine used	Phantom used	Contrast resolution	Spatial resolution (lp/cm)	Acrylic	Air	Bone	Polyethylene	Water	Image scaling	HU uniformity
**Imaging protocol**: Head										
HyperSight	H	1.9	6	115.6	−999.8	932.0	−99.4	−3.4	0.2%	4.32
Somatom Edge	H	1.3	6	123.1	−980.5	854.0	−86.1	1.6	0.4%	0.49
TrueBeam	H	0.6	8	156.1	−998.9	1144.8	−92.0	21.2	0.2%	56.52
Halcyon 2.0	H	0.1	6	76.1	−998.6	1012.1	−141.0	−24.8	0.1%	56.87
**Imaging protocol**: Abdomen										
HyperSight	B	0.4	5	102.7	−973.2	834.3	−95.2	−12.5	0.5%	6.78
Somatom Edge	B	0.5	6	120.8	−961.8	793.7	−79.4	0.2	0.1%	0.53
TrueBeam	B	–	–	–	–	–	–	–	–	–
Halcyon 2.0	B	–	–	–	–	–	–	–	–	–
**Imaging protocol**: Pelvis										
HyperSight	B	0.3	5	102.1	−974.2	838.5	−95.1	−12.5	0.5%	5.31
Somatom Edge	B	0.6	6	124.4	−963.4	798.2	−78.7	1.3	0.0%	0.97
TrueBeam	B	0.2	5	54.3	−958.6	714.7	−126.3	−81.3	0.2%	38.30
Halcyon 2.0	B	0.2	5	78.5	−936.6	795.5	−75.6	−27.2	0.1%	118.82
**Imaging protocol**: Pelvis large										
HyperSight	B	0.7	5	92.0	−962.4	765.9	−99.4	−21.5	0.1%	7.42
Somatom Edge	B	–	–	–	–	–	–	–	–	–
TrueBeam	B	–	–	–	–	–	–	–	–	–
Halcyon 2.0	B	0.1	5	60.7	−917.0	718.0	−88.2	−36.5	0.0%	102.10
**Imaging protocol**: Pediatric head										
HyperSight	H	1.9	6	117.9	−999.9	939.3	−96.2	0.2	0.2%	3.64
Somatom Edge	H	0.6	6	123.6	−980.0	853.7	−85.4	1.9	0.2%	0.77
TrueBeam	H	–	–	–	–	–	–	–	–	–
Halcyon 2.0	H	–	–	–	–	–	–	–	–	–
**Imaging protocol**: Pediatric abdomen										
HyperSight	H	0.6	5	118.4	−998.5	937.9	−96.4	−0.3	0.2%	2.73
Somatom Edge	H	1.0	6	123.5	−982	853.6	−86.5	1.4	0.1%	0.75
TrueBeam	H	–	–	–	–	–	–	–	–	–
Halcyon 2.0	H	–	–	–	–	–	–	–	–	–

*Note*: Machine comparison includes the Varian HyperSight, a diagnostic CT simulator (Siemens SOMATOM Definition Edge), a Varian TrueBeam, and a Varian Halcyon 2.0. All images acquired with 2 mm slice thicknesses (green = meeting ACR criteria, yellow = minor deviation from ACR criteria, red = major deviation from ACR criteria) (H = ACR head phantom, B = ACR body phantom).

Table 4 also provides ACR criteria passing rate comparisons between the HyperSight system and existing linac‐based CBCT imaging systems, a traditional c‐arm Varian TrueBeam 2.7 and a similarly designed ring‐gantry Varian Halcyon 2.0. Huge increases in CNR, HU accuracy and uniformity between the HyperSight and existing linac CBCT imaging systems can be seen. The Head protocols on the HyperSight system are far surpassing the image quality produced by existing CBCT equipment, capable of meeting all ACR criteria compared to 4/9 (44.4%) on a TrueBeam 2.7 and 3/9 (33.3%) on a Halcyon 2.0 (Table 4). The HyperSight Pelvis imaging protocol also outperformed the TrueBeam 2.7 and Halcyon 2.0 with 4/9 meeting no deviation criteria compared to 2/9 (22.2%) on the TrueBeam and Halcyon. The Pelvis Large protocol, available only on the HyperSight system and the Halcyon 2.0 system, has matching passing rates between the two systems; however, the deviation between the produced and ACR passing values for CNR, HU accuracy, and HU uniformity are significantly reduced on the HyperSight system compared to the Halcyon 2.0 system.

**FIGURE 5:**
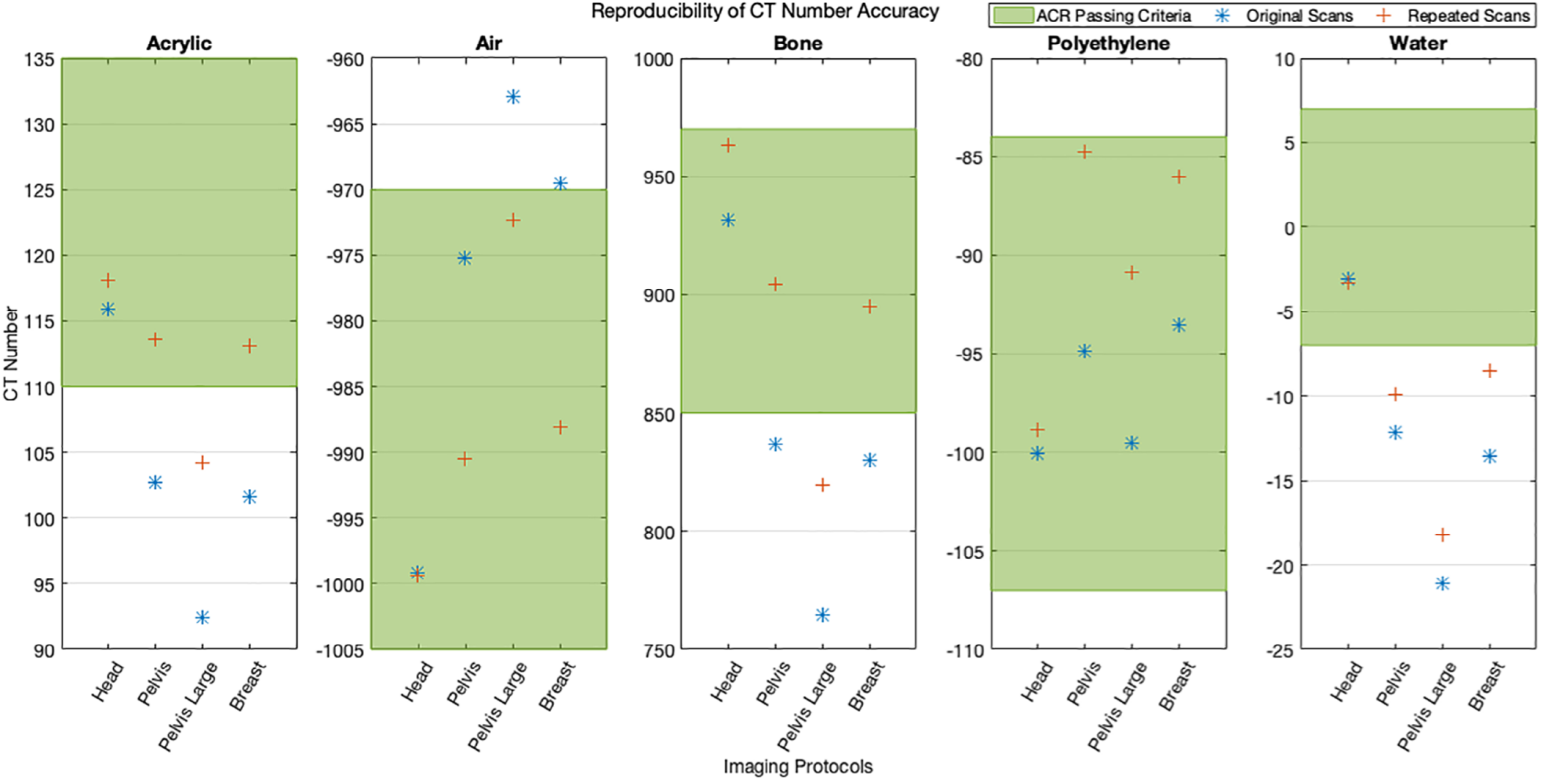
CT number accuracy for four imaging protocols for four HyperSight imaging protocols with a repeated set of scans several months later (green = ACR passing criteria HU range for each material, blue stars = original scan values, red pluses = repeated scan values). Each repeated imaging protocol used the same default imaging parameters with a slice thickness of 3 mm, with the Head protocol using the ACR head phantom and the Pelvis, Pelvis Large, and Breast protocols using the ACR body phantom.

The HU reproducibility analysis results of the Head, Pelvis, Pelvis Large, and Breast imaging protocols can be found in Figure 5, which displays the ACR passing criteria HU regions for each material and the original and repeated scan recorded HU values for each protocol. The Head imaging protocol matched the passing criteria between the repeated scan with the largest reproducibility difference in reported HU values being for the Bone insert (32 HU difference), all other materials had values ≤2.5 HU of the original scans. The repeated Pelvis, Pelvis Large, and Breast imaging protocol scans performed better than the original scans. The original Pelvis scan passed 2/5 materials (failing for acrylic, bone, and water), while the repeated Pelvis scan passed 4/5 materials (failing only water). The Pelvis Large protocol was marginally improved, with the original scan passing 1/5 materials (failing acrylic, air, bone, and water), while the repeated Pelvis Large scan passed 2/5 materials (failing acrylic, bone, and water). The Breast protocol was substantially improved, with the original scan passing only 1/5 materials (failing acrylic, air, bone, and water) and the repeated scan passing 4/5 materials (failing only water). The largest HU differences seen for all imaging protocols between the original and repeated scans was for the Bone material insert, with HU differences of 32, 67.4, 54.9, and 64.9 HU for the Head, Pelvis, Pelvis Large, and Breast protocols. All other materials (acrylic, air, polyethylene, and water) had HU differences of less than 20 HU between the original and repeated scans.

## 4 DISCUSSION

This study provides the first image quality review of the newly introduced Varian HyperSight imaging system, which is the first linac‐based CBCT imaging system with image quality and HU accuracy capable of treatment planning. With the ACR phantom image quality analysis being a widely recognized baseline for diagnostic CT simulators, this study investigates only the image quality achievable by the HyperSight system and provides a benchmark to a diagnostic CT simulator and a comparison to other pre‐existing linac‐based CBCT imaging systems. The accuracy of HU values produced by CBCT for treatment planning is an active area of research,^20^, ^21^, ^22^, ^23^ it has been suggested that HU values within +20 HU near water and + 50 HU near bone is acceptable for treatment planning and dose calculation.^24^

The cause of variations in HU values produced by the HyperSight between several month time periods is not fully known at this time; the HyperSight imager was recalibrated directly before the repeated scans, which may have an impact. However, while the ACR accreditation for HU accuracy is based on HU values falling within a specific range, in clinical practice, the HU accuracy tolerances for kV CBCT images typically have tolerances of ± 50 HU from a baseline value.^25^, ^26^ If baseline values were created for the HyperSight system using the originally scanned HU accuracy values for each material for all scanned imaging protocols (using three slice thicknesses), baseline values of 116.7, −998.6, 931.6, −99.1, and −2.0 would be produced for the ACR head phantom and values of 100.4, −970.2, 814.7, −96.0, and −14.0 HU would be produced for the ACR body phantom. With these baseline values, all but 1 the original scan HU accuracy values would be within the suggested ± 50 HU tolerance (Pelvis Large bone material fails at ± 50.2 HU; meanwhile the repeated scans have both the Pelvis and Breast imaging protocols failing the bone material HU accuracy tolerance with values of ± 89.7 HU and ± 80.4 HU.

The CBCTp mode images produced by the Varian HyperSight are of excellent quality, rivaling those produced by traditional diagnostic CT simulators, and far surpassing CBCT protocols available on current linacs. The major CBCT image quality improvements observed are increased CNR, HU accuracy, and HU stability and uniformity, which can be attributed to the upgraded hardware and software modifications within the HyperSight package discussed previously. While the HU accuracy and stability displayed within this study by the HyperSight CBCT system is far surpassing other CBCT systems presently in clinical use, an in‐depth analysis on the HU accuracy of the HyperSight system is warranted and will be investigated in later works.

One limitation of this study was that all images were acquired using only the default settings of each vendor‐provided imaging protocol, with the automatic exposure (AEC) setting turned off. Pediatric Head and Pediatric Abdomen protocols were tested on ACR criteria using increased, non‐default exposure settings to confirm the stability of passing rates; however, no images or passing rate tests were done using exposure settings lower than the vendor‐provided default settings. It is acknowledged that using exposures lower than the default setting, or using the AEC setting, may result in reduced image quality. Additionally, HyperSight users can create new imaging protocols, which may result in increased or reduced image quality depending on the imaging dose the user prefers. Therefore, each institution and user may receive slightly different levels of image quality, with the potential that not all lower‐dose imaging may be capable of passing ACR criteria that match the quality produced by diagnostic CTs.

## 5 CONCLUSIONS

The Varian HyperSight is a ring gantry linac‐mounted advanced kV‐CBCT imaging system designed for daily image guidance (IGRT mode) and radiation therapy treatment planning (CBCTp mode). This study investigated its CBCTp mode capabilities in passing the ACR diagnostic CT image quality standards using the head and body ACR phantoms. We also compared HyperSight CBCTp mode passing rates with a traditional diagnostic CT scanner and two types of existing linac‐based CBCT systems (Halcyon version 2.0 and TrueBeam 2.7). The HyperSight system produces CBCT images of excellent quality, sufficient for treatment planning purposes. All vendor‐provided default imaging protocols on the HyperSight system can pass all ACR criteria for CNR, spatial resolution, HU accuracy, and image scaling, and almost all protocols pass HU uniformity criteria, with only three protocols producing minor deviations on HU uniformity (5–7 HU maximum difference between center and peripheral ROIs). The larger diameter body phantom ring caused slightly more HU accuracy and uniformity deviations from ACR criteria, resulting in ACR criteria minor failures in CNR, acrylic HU accuracy, air HU accuracy, bone HU accuracy, water HU accuracy, and HU uniformity, but still produced overall very good quality images. When compared to a diagnostic CT simulator, the HyperSight system's vendor‐provided imaging protocols almost identically matched the ACR passing rates for matching default imaging protocols at similar CTDIvol levels. The HyperSight system had slightly less HU uniformity, with one imaging protocol producing a minor deviation (5.3 HU), but also produced higher CNR compared to the diagnostic CT for nearly all default protocols. When compared to existing linac‐based CBCT systems, the HyperSight system far exceeded the image quality and ACR passing rates of a Varian TrueBeam 2.7 and a Varian Halcyon 2.0. This is a first step towards the future application of HyperSight CBCT images directly for contouring and treatment planning without a companion diagnostic CT scan.

## AUTHOR CONTRIBUTIONS

Taoran Li, Boon‐Keng Teo, Lei Dong designed the study, Allison Haertter, Michael Salerno, Brandon Koger performed key measurements and data analysis, AH led the manuscript drafting, Christopher Kennedy and Michelle Alonso‐Basanta provided important input during data processing and writing process. All authors contributed substantially to the overall manuscript.

## CONFLICT OF INTEREST STATEMENT

This work was, in part, financially sponsored by Varian Medical Systems. Investigators independently designed and performed the study.
